# Melatonin: A Mitochondrial Targeting Molecule Involving Mitochondrial Protection and Dynamics

**DOI:** 10.3390/ijms17122124

**Published:** 2016-12-16

**Authors:** Dun-Xian Tan, Lucien C. Manchester, Lilan Qin, Russel J. Reiter

**Affiliations:** Department of Cell System and Anatomy, The University of Texas Health Science Center, San Antonio, TX 78229, USA; lmanchester@stmarytx.edu (L.C.M.); qinl@uthscsa.edu (L.Q.)

**Keywords:** melatonin, antioxidant, mitochondria, mitophagy, mitochondrial dynamics

## Abstract

Melatonin has been speculated to be mainly synthesized by mitochondria. This speculation is supported by the recent discovery that aralkylamine *N*-acetyltransferase/serotonin *N*-acetyltransferase (AANAT/SNAT) is localized in mitochondria of oocytes and the isolated mitochondria generate melatonin. We have also speculated that melatonin is a mitochondria-targeted antioxidant. It accumulates in mitochondria with high concentration against a concentration gradient. This is probably achieved by an active transportation via mitochondrial melatonin transporter(s). Melatonin protects mitochondria by scavenging reactive oxygen species (ROS), inhibiting the mitochondrial permeability transition pore (MPTP), and activating uncoupling proteins (UCPs). Thus, melatonin maintains the optimal mitochondrial membrane potential and preserves mitochondrial functions. In addition, mitochondrial biogenesis and dynamics is also regulated by melatonin. In most cases, melatonin reduces mitochondrial fission and elevates their fusion. Mitochondrial dynamics exhibit an oscillatory pattern which matches the melatonin circadian secretory rhythm in pinealeocytes and probably in other cells. Recently, melatonin has been found to promote mitophagy and improve homeostasis of mitochondria.

## 1. Introduction

Mitochondria are important organelles in eukaryotes. They are referred to as the powerhouse of the cell since adenosine triphosphate (ATP), a source of chemical energy that sustains the biological activities and development of the cells, is mainly generated by mitochondria. Based on the endosymbiotic theory proposed by Sagan [1], mitochondria are probably derived from primitive photosynthetic bacteria. When a relatively large protoeukaryotic cell engulfed a smaller photosynthetic bacterium, the host cell did not digest it, but the photosynthetic bacterium parasitized the host. The host provided ample resources, such as carbohydrates and amino acids, to the parasitic bacterium; in turn, the bacterium rewarded the host with more ATP. Thus, they were mutually beneficial. During evolution, the parasitic bacterium evolved to be the mitochondrion and become an essential organelle of the host cell. The bacterial characteristics of mitochondria appear to be partially preserved. For example, they still retain bacterial cyclic DNA. In addition, mitochondria and mitochondrial DNA can be horizontally transferred from cell to cell in mammalian cell culture systems and in plants [2,3,4]. There is, however, no definitive evidence to show whether this mitochondrial movement occurs in vivo in animals. The intercellular mitochondrial transfer should be considered a fundamental physiological process with a role in development and tissue homeostasis. Mitochondria are multifunctional organelles. They contribute to cellular calcium homeostasis, trigger apoptosis, and regulate cellular metabolism [5,6,7,8]. However, the primary function of mitochondria is to generate ATP to power cells.

During ATP production, the electrons captured by the electron transporters including the coenzyme Q (CoQ) and cytochrome C eventually are transported to oxygen to form water. This process occurs in the electron transport chain (ETC) localized in the inner membrane of the mitochondria. Some electrons inevitably leak from the ETC and incompletely reduce oxygen to form free radicals, mainly the superoxide anion (O_2_•^−^). O_2_•^−^ is an essential signaling molecule for cellular functions [9,10]. However, its excessive production results in oxidative stress and cellular injury, which may lead to cell death. O_2_•^−^ can be autodismutated or via dismutase to form hydrogen peroxide (H_2_O_2_). H_2_O_2_ has a much longer half-life than that of O_2_•^−^. This allows H_2_O_2_ to diffuse to other cellular compartments to induce wide ranging oxidative stress [11]. The worst case is the homolysis of H_2_O_2_ (such as in the Fenton or Haber–Weiss reactions) to generate the hydroxyl radical (HO•); this is the most reactive and disreputable free radical [12]. There is no enzyme to detoxify HO• since its turnover rate is in the nanosecond range. As a result, it injures macromolecules including lipids, proteins, DNA, and carbohydrates in its vicinity [13,14].

O_2_•^−^, H_2_O_2_, HO• and other oxygen-related species are collectively referred to as the reactive oxygen species (ROS); when nitrogen is involved, such as nitric oxide (NO•) and peroxynitrite (ONOO^−^), they are called reactive nitrogen species (RNS). Fortunately, cells have developed strategies to protect against oxidative stress induced by both ROS and RNS [15,16]. One of the mechanisms involves melatonin. Melatonin is classified as a potent free radical scavenger and a mitochondrial-targeted antioxidant [17,18,19,20]. Melatonin scavenges a broad spectrum of ROS and RNS, especially, the HO• [21,22,23]. High levels of melatonin compared to other cellular compartments have been identified in mitochondria [24,25,26,27,28]. A variety of in vitro and in vivo studies have proven that melatonin targets mitochondria to reduce oxidative stress [29,30,31,32,33,34,35,36]. This results in decreased apoptosis, improved metabolic status, and an elevated survival rate of cultured cells, unicellular organisms, animals, and plants, which suffer with oxidative stress [37,38,39,40,41,42,43,44,45]. The mechanisms of melatonin as a mitochondrial protector not only relate to its excellent free radical scavenging capacity but also to its function as a signaling molecule to upregulate gene expression of antioxidant enzymes [46,47,48] and a spectrum of stress responsive genes [49,50,51,52,53,54,55,56]. In addition, melatonin acts on the mitochondrial specific proteins such as uncoupling proteins (UCPs) to dissipate the proton gradient across the inner membrane of the mitochondria to moderately reduce the inner membrane potential [57,58,59,60]. The relative lowering of the inner membrane potential significantly increases the activities of complex I and III, and accelerates electron transport through the ETC. These changes decrease electron leak from the ETC and reduce free radical formation. This is referred to as the free radical avoidance reaction of melatonin [61]. Not only melatonin per se but several of its metabolites including 2-hydroxylmelatonin, 6-hydroxylmelatonin, cyclic 3-hydroxymelatonin, *N*^1^-acetyl-*N*^2^-fomyl-5-methoxykynuramine (AFMK), and *N*^1^-acetyl-5-methoxykynuramine (AMK) are also antioxidants [20,62,63,64,65,66,67,68]. Cyclic 3-hydroxymelatonin and AMK were reported to be more potent than melatonin toward their reaction with ROS [69,70,71,72,73].

In plants, 2-hydroxymelatonin is a major melatonin metabolite, and its level is two orders of magnitude higher than that of melatonin [74]. This metabolite may have greater efficiency to reduce plant abiotic stress than that of melatonin [75]. This phenomenon may also exist in animals [76]. The continuous free radical scavenging activities of melatonin and its metabolites are referred to as a free radical scavenging cascade reaction [15,77]. The cascade reaction of melatonin renders it an excellent antioxidant. Herein, the potential association of melatonin with mitochondria is considered.

## 2. Mitochondria: The Major Sites for Melatonin Synthesis and Metabolism

The pineal gland initially was considered the exclusive organ in vertebrates that produced and secreted melatonin [78]. Pinealocytes, astrocytes, and microglia are the main cells of the gland [79,80,81]. The expression of *AANAT* gene has been identified in astrocytes, and both astrocytes and microglia were reported to synthesize melatonin [82,83]. It is presumed that the major portion of melatonin released from the pineal gland to circulation is produced by the pinealocytes. The physiological function of the astrocytes and microglia in the pineal gland may be to support melatonin synthesis in pinealocytes. The paracrine modulation of melatonin synthesis in pinealocytes by astrocytes and microglia seems to be a basic network. The network is initiated by the activation of nuclear factor κB (NF-κB) in astrocytes and microglia by different stimuli. These cells then release tumor necrosis factor (TNF), which signals pinealocytes to synthesize melatonin [84,85,86].

Here, we need to address the so-called physiological level of melatonin. The physiological level of melatonin in serum of mammals is in the range of 10^−9^ M. However, the physiological levels of melatonin in different tissues, organs, or cells seem considerably higher than that in serum [28]. For example, the physiological level of melatonin in the pineal recess of the third ventricle of sheep is at least 100-fold higher than that in the serum [87]. In unicellular organism, the physiological levels of melatonin reach 10^−4^ to 10^−3^ M [88]. As a result, it is difficult to distinguish the “physiological” levels of melatonin from pharmacological values depending on the tested fluid or tissue.

**Figure 1:**
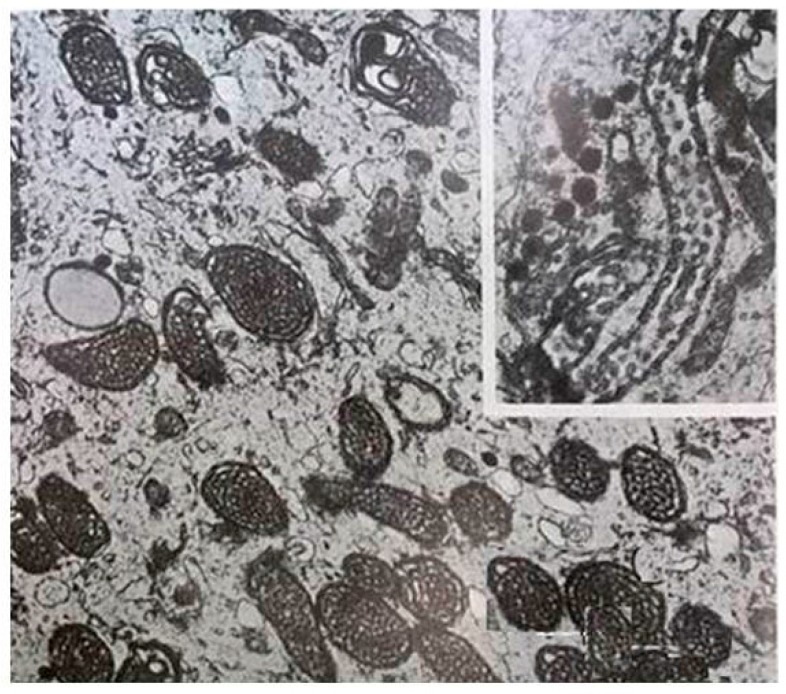
Large amounts of mitochondria are present in pinealocytes of the Syrian hamster (34,000×). Inset shows a longitudinal section of mitochondrion with cristae arranged like a string of beads (44,500×). Modified from Bucana et al. [89].

It was recognized decades ago that the cytoplasm of pinealocytes is rich in mitochondria [89,90,91] (Figure 1). The mitochondrial density in pinealocytes is several-fold higher than that in neurons. This phenomenon cannot be simply explained by the metabolic rate of pinealocytes since there is no evidence to show that their metabolic rates are higher than that of neurons. In addition, the morphology of the mitochondria in pinealocytes changes dynamically with the light/dark cycle as well as with the activity of the pinealocytes in different species [91,92,93,94]. During the dark period, corresponding with the melatonin synthetic peak, there are greater relative volumes of mitochondria in pinealocytes compared to the daytime [92]. When male mice were exposed to constant light, not only was melatonin production depressed, but many pinealocyte mitochondria appeared swollen with a rarified matrix and reduced numbers of cristae [95]. These changes suggest that an additional function of mitochondria, besides ATP production, may be associated with melatonin synthesis. Interestingly, Kerenyi et al. observed that the reaction product of AANAT was exclusively localized in the mitochondria of mouse pinealocytes [96,97]. These authors failed to explain the potential significance of their observations; therefore, their reports did not draw the attention of pineal scientists. It is our belief that, in addition to pinealocytes almost all organs, tissues and cells have the capacity to synthesize melatonin [28,98]. Thus, while pinealocytes are differentiated to be specific cells which produce melatonin, many other cells, no matter their location and type, may still have melatonin synthetic capacity. Different from the pinealocytes where melatonin is released into the blood and cerebrospinal fluid (CSF) as a signaling molecule to convey photoperiodic information [87,99], melatonin synthesized by other cells is presumably used locally for defense against oxidative stress and inflammation [100].

Melatonin is already present in unicellular organism, e.g., algae [88,101] and is also present in photosynthetic bacteria such as *Rhodospirillum rubrum* [102], *Erythrobacter longus* [103], and cyanobacteria [104]. We have speculated that its origin can be traced to almost 2.5 billion years ago, when the photosynthetic bacteria such as *Rhodospirillum rubrum* and cyanobacteria thrived [105]. *Rhodospirillum rubrum* is considered as the close precursor of mitochondria [106], and so are the cyanobacteria as the precursors of chloroplasts [107]. We hypothesized that the melatonin synthetic capacity of these bacteria was horizontally transferred to the eukaryotes. Thus, mitochondria inherited the melatonin synthetic capacity from the α-proteabacteria and chloroplasts inherited this capacity from cyanobacteria [108]. This hypothesis has been supported by the observations of Byeon et al. [109]. They reported that in red alga (*Porphyra yezoensis*), the genome of chloroplasts encodes the *SNAT* gene, which is the rate limiting enzyme in melatonin synthesis in plants. Phylogenetic analysis of the sequence suggested that the *SNAT* encoded in chloroplasts of *Porphyra yezoensis* evolved from the cyanobacteria *SNAT* gene via endosymbiotic gene transfer roughly 1.5 billion years ago. The red alga appears to be the transit species since their chloroplasts hold the *SNAT* gene; sometime thereafter, the melatonin synthetic genes in other species were incorporated into the nuclear DNA from the chloroplast genome. However, the status of chloroplasts as a major site for melatonin synthesis remains unchanged. The *SNAT* encoded in the nucleus requires a chloroplast transit peptide to re-enter the chloroplast. The evolution of these transit peptides have been predicted in other species [109]. This indicates that this melatonin synthetic enzyme encoded by the nucleus was transported to the chloroplasts. Indeed, in rice and in *Arabidopsis*, the SNAT protein was identified to be localized in chloroplasts [110]. This provides direct evidence to show that chloroplasts are the site for melatonin production, especially if the final step of melatonin synthesis is acetylation of 5-methoxytraptime, which is hypothesized to be carried out by SNAT. This revised pathway of the classic route was predicted to be dominant in plants and perhaps in animals [76].

**Figure 2:**
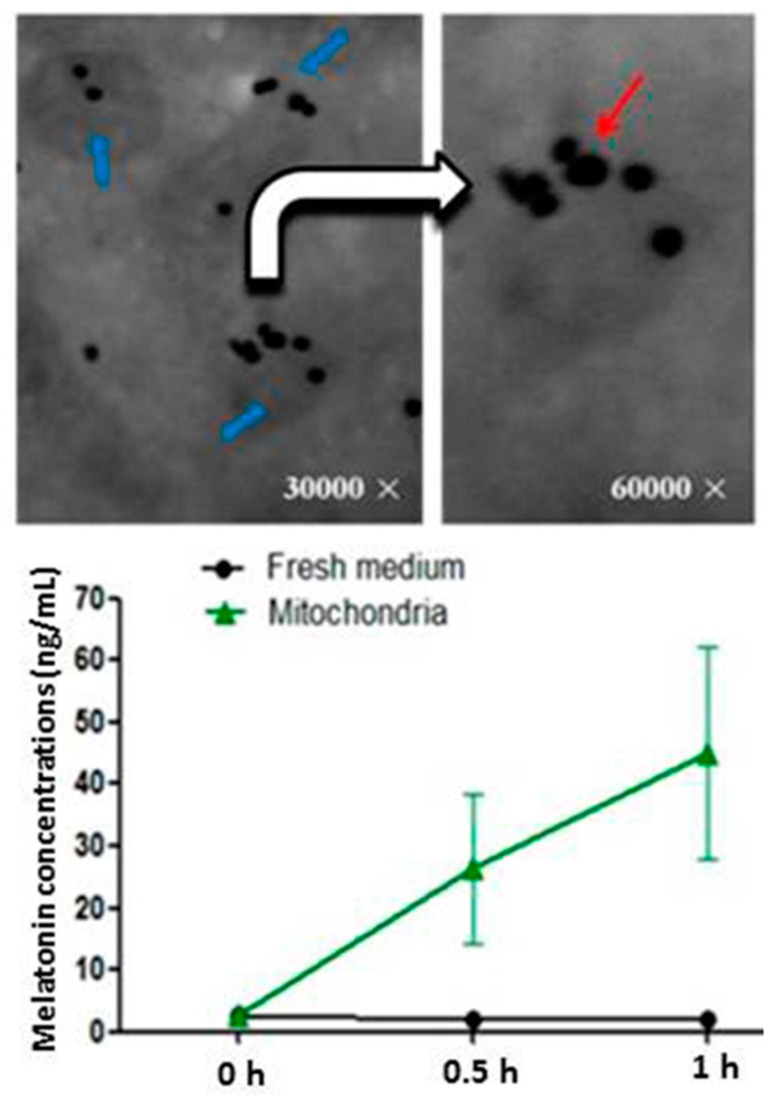
**Upper** panel: The localization of the SNAT. Blue arrows: The mitochondria isolated from the oocytes of mice. White arrow: enlarged image from left side of the arrow tail. Red arrow: SNAT staining (black dot). SNAT: serotonin *N*-acetyltransferase; **Low** panel: Melatonin concentrations in mitochondrial culture media with 10^−4^ M serotonin (mean ± SEM) modified from He et al. [112].

As to mitochondria, several lines of evidence indicate their ability for melatonin synthesis. The much higher levels of melatonin in these organelles have been reported [27,28]. The mitochondrial melatonin level was roughly 100-fold higher than that in the plasma of mice [111]. Moreover, the products of aralkylamine *N*-acetyltransferase/serotonin *N*-acetyltransferase AANAT/SNAT are found to be exclusively present in the mitochondria of pinealocytes. The production of melatonin is reflected in the morphological alterations of the mitochondria in pinealocytes. While this is indirect evidence, the direct evidence comes from the recent observations of He et al. that AANAT is confined to the mitochondria of oocytes of mice [112] (Figure 2). The genes for melatonin synthetic enzymes are expressed in oocytes, and these cells also synthesize melatonin [113,114]. It appears that melatonin in oocytes may be predominantly produced in the mitochondria. When the isolated mitochondria from the oocytes were cultured in medium with tryptophan, significantly higher levels of melatonin were detected in the culture medium compared to those in the control media (Figure 2).

Interestingly, mitochondria seem not only to synthesize melatonin but also to metabolize it. The melatonin metabolite, AFMK, was detected in mitochondria. Cytochrome C is believed to participate in this melatonin metabolic process [115]. This is not surprising since cytochrome C is a conserved molecule and is present in the photosynthetic bacteria [116]. We thus speculate that, in bacteria, cytochrome C functions as an ancient process to metabolize melatonin. This function of cytochrome C is preserved in the mitochondria of present-day species. The potential mechanism as to how cytochrome C converts melatonin to AFMK lies in its heme iron. This was discussed in a previous publication [100].

That melatonin is synthesized in mitochondria does not exclude the possibility of melatonin also being synthesized in the cytosol. Melatonin synthesis in cytosol has been a mainstream concept and AANAT/SNAT is also found in the cytosolic compartment. However, judging from the kinetics of AANAT/SNAT and the substrate availability, it is obvious that cytosolic melatonin synthesis is far less efficient than that in mitochondria. A direct substrate of AANAT/SNAT is acetyl CoA. This substrate is mainly produced in mitochondria. The calculated *K*_m_ of AANAT for acetyl CoA is 0.11 ± 0.02 mM under a fixed tryptamine concentration of 10 mM [117]. The estimated acetyl CoA concentration in mitochondria is around 0.5–1.0 mM [118], and the estimated cytosolic acetyl CoA concentration is 3–30 µM [119]. The concentrations of acetyl CoA in other cellular compartments such as in the cytosol are far below the *K*_m_ of the AANAT; however, the concentration of acetyl CoA in mitochondria can satisfy the *K*_m_ of the AANAT. From an enzymatic kinetics and available substrate point of view, mitochondria with a suitable concentration of acetyl CoA are likely the most important site for melatonin synthesis in organisms.

## 3. Melatonin: A Potent Protector of Mitochondria

Functional mitochondria decide the fate of the cells. Not only do the mitochondria provide the biochemical energy to power the basic activities of the cell but, mitochondria can initiate the death signal for apoptosis. To preserve mitochondrial morphology and function is important for healthy cells. Many mitochondrial-targeted agents have been synthesized and tested for this purpose [120]. However, not all of them have produced the expected results. One of the major obstacles is the mitochondrial permeability of these agents. Mitochondrial membrane has a limited permeability for many substances. In most cases, the transmembrane transporters are required to carry molecules into mitochondria. The successful synthetic agents include mitochondrial-targeted Coenzyme Q10 (MitoQ) and mitochondrial-targeted vitamin E (MitoE) in which the active antioxidant moieties are covalently coupled to a lipophilic triphenylphosphonium cation. MitoQ and MitoE can accumulate several-hundred fold within the mitochondrial matrix, driven by the organelle’s large membrane potential [121,122]. The protective effects of these substances against cell damage have frequently been reported, and clinical applications are implicated [123,124,125].

A comparison of these synthetic agents with naturally occurring melatonin was made in a septic shock mouse model [126]. The results showed that melatonin was even more efficient than the artificially produced mitochondrial-targeted antioxidants, MitoQ and MitoE, regarding cellular protection. It is presumed that this protective effect requires high levels of melatonin accumulation in mitochondria.

What are the mechanisms by which melatonin can accumulate in mitochondria against a concentration gradient? Even through melatonin is a lipophilic molecule and can cross the plasma membrane with ease, the passive diffusion of melatonin cannot explain this phenomenon. Recently, it was reported that glucose transporter 1(GLUT1) may also transport melatonin into cells, and this function is dependent on glucose levels [127]. Whether this transporter functions for the transfer of melatonin into mitochondria is unanswered. A recent study, however, reported that the peptide transporters 1 and 2 (PEPT1/2), also known as solute carrier family 15 members 1 and 2 (SLC15A1/2), are localized in the mitochondrial membrane and are responsible for melatonin transport into mitochondria (unpublished observation, Ma et al.). The expression levels of these transporters in mitochondria were positively associated with the concentrations of mitochondrial melatonin. The presence of PEPT1/2, and likely other transporters, in mitochondria are probably responsible for melatonin transport into this organelle. This active transport causes mitochondrial melatonin accumulation and provides cellular protection.

The first evidence of melatonin as a mitochondrial protector came from the report of Mansouri et al. [128]. The authors reported that melatonin attenuated the ethanol-induced hepatic mitochondrial DNA depletion in mice with the mechanism being related to melatonin’s antioxidant capacity. Martin et al. [25] subsequently observed that melatonin prevented the inhibition of mitochondrial complexes I and IV induced by ruthenium red and significantly reduced mitochondrial oxidative stress caused by *t*-butyl hydroperoxide; however, comparable doses of vitamins C and E lacked these protective effects [111]. The differences among melatonin and vitamin C and vitamin E on the relative protection of mitochondria may be explained by the observations that melatonin accumulates in mitochondria perhaps via the active transport by PEPT1/2 (unpublished observations Ma et al.), but this is not the case with vitamin C and E. Many studies have confirmed the protective effects of melatonin against mitochondrial injury caused by different insults including ischemia/reperfusion [129,130], sepsis [131,132,133], in vitro fertilization (IVF) [134,135,136,137], 1-methyl-4-phenylpyridinium ion (MPP^+^) [138], β-amyloid peptide (Aβ 25–35) [139,140], rotenone [141], 4-hydroxynonenal [142], arsenite [143], and lipopolysaccharide [144]. In addition to these mitochondrial injuries induced by exogenous interventions, melatonin also exhibits significant beneficial effects on several neurodegenerative diseases related to mitochondrial dysfunctions per se. These include Huntington’s disease (HD), which is an autosomal dominant neurodegenerative disorder where the alterations in mitochondrial function play a key role in the pathogenic processes [145]. Melatonin administration significantly delayed disease onset and mortality in a transgenic mouse model of HD [146]. Interestingly, in this report, the melatonin receptor 1 (MT1) was, for the first time, identified in the mitochondrial membrane. The mitochondria in the transgenic mouse model of HD contain many fewer MT1 than that of the wild type. The authors concluded that one of the etiologies of HD was the loss of the mitochondrial MT1 receptor, leading to an enhancement of neuronal vulnerability that potentially accelerates this neurodegenerative process.

Multiple sclerosis (MS) is the most prevalent inflammatory demyelinating disease of the central nervous system. Mitochondrial abnormalities including mitochondrial genetic alterations, mitochondrial enzyme disability, and faulty mitochondrial DNA repair contribute to the progress of this disease [147]. In a mouse model of MS, melatonin treatment prevented the pathological alterations by restoring mitochondrial respiratory enzyme activity and fusion and fission processes as well as by reducing intra-axonal mitochondria accumulation [148]. Moreover, a recent report showed that the treatment of a patient suffering with primary progressive MS exhibited significant clinical improvement after low-dose melatonin treatment [149]. The protective mechanisms of melatonin on mitochondria are multiple. These include, but are not limited to, a reduction of mitochondrial oxidative stress [150,151], preservation of the mitochondrial membrane potential [152,153,154], upregulation of the antiapoptotic mitochondrial protein/downregulation of the proapoptotic mitochondrial protein, Bax [137,155,156], increased efficiency of ATP production [59,157], reduced release of cytochrome C into the cytosol and the inhibition of caspase 3 activity [158,159].

Many studies have addressed the importance of melatonin’s effects on the mitochondrial membrane potential (Δψ). This potential is important for ATP generation and for maintaining the complete function of mitochondria. The mitochondrial permeability transition pore (MPTP) plays a critical role in preserving the optimal Δψ. Induction of the MPTP increases mitochondrial membrane permeability to molecules of less than 1500 Daltons in molecular weight and causes mitochondria to become further depolarized, leading to the Δψ collapse, cytochrome C release, mitochondrial swelling, and cellular apoptosis. The MPTP inhibitor, cyclosporine, an immunosuppressive agent, reduces Δψ collapse and the resulting cellular apoptosis. The mechanism is that cyclosporine binds to the cyclophilin D protein (CypD), which constitutes part of the MPTP to block the calcium flashing into the mitochondria [160,161], and inhibits the calcineurin phosphatase pathway [162]. Melatonin is also a MPTP inhibitor, but with a different mechanism. It has been documented that the ADP/ATP carrier (AAC) can also serve as the MPTP. Normally, AAC is closed due to its tight binding to cardilipin [163]. The prooxidation of the bond cardilipin results in AAC configuration modification to its open form which induces calcium overload and Δψ collapse. Melatonin as a mitochondrial antioxidant protects cardilipin from pro-oxidation and therefore maintains the closed configuration of AAC. The protective effects of melatonin on cardilipin and MPTP are well documented [164,165,166,167].

Other structures that are associated with mitochondrial membrane potential are uncoupling proteins (UCPs). Different from the MPTP, UCPs can be actively regulated by many factors based on the status of the mitochondria. Activation of UCPs usually has beneficial effects on mitochondrial functions including balancing the Δψ, accelerating electron transport and finally reducing ROS formation and cellular oxidative damage [168,169]. Melatonin increases the activity of UCPs either by upregulating gene expression or directly acting on these proteins [57,58,60]. The activation of UCPs shuttles the intermembrane protons back to the matrix and slightly reduces the Δψ. The relatively lowered Δψ accelerates electron transport in the ECT; therefore, electron leakage is dramatically decreased, as is ROS formation. This function of melatonin may be more significant than its direct free radical scavenging action [61]. Theoretically, activation of the UCPs results in the uncoupling of oxidative-phosphorylation and a decrease in ATP production. However, ATP production is not compromised by melatonin’s effect on UCPs. The potentially reduced ATP production caused by the activation of UCPs may be counteracted by the fewer leaked electrons (which carry energy) and accelerated electron transportation induced by melatonin, since several studies have reported that melatonin increase the ATP production under different conditions [59,112,157,170,171,172,173,174,175].

## 4. Melatonin Regulates Mitochondrial Dynamics

**Figure 3:**
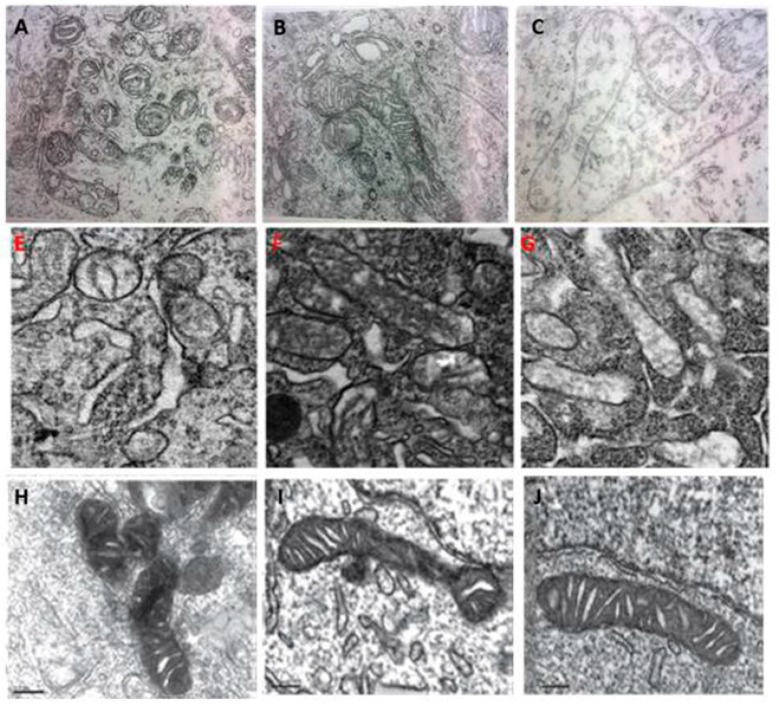
The similarities of mitochondrial dynamics in pinealocytes, brain neurons and cultured SH-SY5Y cells. **Upper** panel: Mitochondrial dynamics in pinealocytes (27,000×). (**A**) Condensed state; (**B**) Second intermediate state; (**C**) Third intermediate state; **Middle** panel: Mitochondrial dynamics in brain neurons of mice. (**E**) Mitochondrial fission induced by cadmium treatment; (**F**) The transition of mitochondrial fission and fusion in the animal treated with cadmium plus melatonin; (**G**) Mitochondrial fusion in control healthy animal; **Lower** panel: Mitochondrial dynamics in cultured SH-SY5Y cells (60,000×). (**H**) Mitochondrial fission induced by methamphetamine; (**I**) The transition of mitochondrial fission and fusion in cells treated with methamphetamine plus melatonin; (**J**) Mitochondrial fusion in control cells. The similarities of **A**, **E** and **H**; **B**, **F** and **I**; **C**, **G** and **J** are obvious. Mordified from [90,184,185].

The functions of mitochondria exhibit significant circadian rhythms, which help mitochondria to cope with alterations in nutrient availability, energy supply, and cellular remodeling, that naturally occur throughout the day. This also involves mitochondrial biogenesis, fission, fusion, and mitophagy. All these maintain mitochondrial and cellular functions. Collectively, these processes are referred to as mitochondrial dynamics [176,177]. The daily oscillations of mitochondria are believed to be dependent on the clock proteins Period1 and Period2 (PER1/2) since they are blunted in mice lacking these proteins [178]. PER1/2 are well known to be regulated by melatonin [179,180]. For example, melatonin induces a rise in the expression of PER1/2 [181]. The regulation of PER1/2 by melatonin not only occurs centrally in the suprachiasmatic nucleus (SCN) but also peripherally, that is, it occurs in peripheral cells [182]. This provides an opportunity for melatonin to directly regulate the mitochondrial oscillations via PER1/2 which are present in peripheral cells [183]. This is supported by the daily changes of mitochondrial morphology which is well coordinated with the melatonin synthetic peak in pinealocytes [91,93]. By carefully studying the daily morphological changes of mitochondria in pinealocytes, Krakowski and Cieciura [90] identified three types of mitochondrial configurations, that is, a condensed state, the second intermediate state, and the third intermediate state (Figure 3A–C). These three states of mitochondria were rhythmically changed over a 24 h period in pinealocytes. This is apparent in the comparison with mitochondrial images obtained from the recent publications that the observations of Krakowski and Cieciura [90] may be the first evidence to show that the mitochondrial biogenesis (fission/fusion) in pinealocytes exhibits oscillations throughout the day. (Figure 3).

The condensed state resembles mitochondrial fission; the second intermediate state is similar to the transit of mitochondrial from fission to fusion; the third intermediate state represents mitochondrial fusion. Since the pinealocytes may specifically synthesize melatonin, these alterations of mitochondria are more than likely regulated by the melatonin concentrations in these cells. Most notably, the mitochondrial fusion (third intermediate state) was always accompanied by the melatonin secretory peak either in the 12/12 h light exposure or in constant darkness conditions. It is difficult to distinguish whether the fused mitochondria produce more melatonin or the high level of melatonin promotes mitochondrial fusion in this study. The point is that mitochondrial biogenesis exhibits a strong association with the melatonin circadian rhythm in pinealocytes. In addition to pinealocytes, melatonin was also reported to regulate the mitochondrial fission/fusion in other cell types [186,187]. The mitochondrial fission/fusion machinery is involved in generating young mitochondria, while eliminating old, damaged, and non-repairable ones. Fission is generally related to the cellular injury and apoptosis and fusion is associated with healthy cells. Under most conditions, an elevated melatonin concentration results in decreased mitochondrial fission but elevated mitochondrial fusion [138,184,185,186,187,188,189]. Mechanistically, melatonin attenuates the mitochondrial translocation of mitochondrial fission proteins mitochondrial fission 1 protein (Fis1), dynamin-related protein 1 (Drp1) and the pro-apoptotic protein, Bax, as well as upregulating mitochondrial fusion proteins (mitofusins 1 and 2 (Mfn1/2)) and optic atrophy 1 (Opa1). Most Drp1 is soluble in the cytosol of cells from where it attaches to the mitochondrial outer membrane [190] where it binds with Fis1. The Drp1 complex assembles into spirals at division sites around the outer mitochondrial membrane to drive the fission process [191]. Melatonin suppresses the translocation of Fis1 and Drp1 to the outer mitochondrial membrane, thus reducing fission. The mechanisms by which melatonin regulates mitochondrial fusion proteins is highly complex. Melatonin may upregulate the expression of *Mfn1* via Notch1 signaling [192] or it could downregulate *Mfn1* and *Opa1* [187]. More studies are required to clarify these processes.

Mitochondrial biogenesis also requires mitophagy. Mitophagy is an autophagic process specifically targeting mitochondria. It cleans up the damaged and non-repairable mitochondria and preserves healthy ones. This process plays a crucial role in the wellbeing of cells, since their autophagic delivery to lysosomes is the major degradative pathway in mitochondrial turnover [193]. The association of melatonin with autophagy is well documented. Majority of the studies report that melatonin suppresses autophagy in cells and organisms which are exposed to different stressors, therefore reducing their injury and improving their recovery. Some reports document that melatonin may also induce or enhance autophagy [143,194,195,196,197,198,199]. The influence of melatonin on autophagy seems well conserved since this association also has been found in plants [200,201]. Based on published data, Coto-Montes et al. [202] speculated that a specific autophagy, i.e., mitophagy, could also be influenced by melatonin. This speculation is supported by the observations summarized herein that melatonin indeed targets the process of mitophagy. Melatonin mainly enhances mitophagy and improves mitochondrial biogenesis [203,204,205]. The exact mechanisms by which melatonin targets mitophagy are not currently available. It seems that this process is mediated by melatonin receptors that activate adenosine 5′-monophosphate-activated protein kinase (AMPK). An activation of AMPK suppresses the mammalian target of rapamycin (mTOR) pathway and elicits mitophagic responses, while AMPK initiates mitochondrial biogenesis via sirtuin1 (SIRT1) dependent deacetylation of peroxisome proliferator-activated receptor γ coactivator 1-α (PGC-1α) or upregulation of PGC-1α expression [203].

## 5. Conclusions

**Figure 4:**
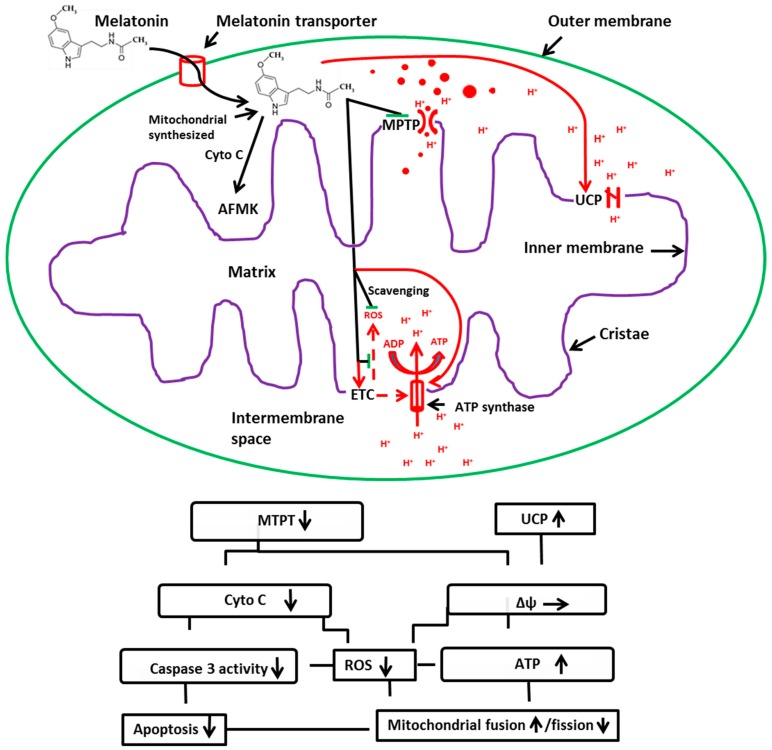
A summary of the potential effects of melatonin on a mitochondrion. MPTP: mitochondrial permeability transition pore; UCP: uncoupling protein; ROS: reactive oxygen species; ETC: electron transport chain; Cyto C: cytochrome C; AFMK: a melatonin metabolite, *N*^1^-acetyl-*N*^2^-fomyl-5-methoxykynuramine, which is also a potent antioxidant. Melatonin is metabolized to AFMK by cytochrome C via pseudo-enzymatic process [77]. **Upper** panel: The targeting sites of melatonin on mitochondrion; green lines: inhibition; red arrows: activation; red dash arrows: the directions of the multiple steps of reactions; black arrows: directions; **Lower** panel: Summary of the outcomes induced by melatonin’s action on mitochondrion; upward arrowhead: activation; downward arrowhead: inhibition; horizontal arrowhead: preservation; connecting lines indicate hierarches of the events.

Mitochondria are important organelles. They not only provide the chemical energy to power the cell, but also regulate cellular homeostasis of calcium, apoptosis, and cellular metabolism. Preservation of the structural and functional integrity of mitochondria is essential for a healthy cell. One of the mitochondrial-targeted molecules is melatonin. Melatonin may be synthesized by mitochondria, a capacity that was inherited from bacteria, the precursors of mitochondria. As a result, all cells with mitochondria likely have the capacity to produce melatonin. This is strongly supported by the observations that the products of AANAT are exclusively located in mitochondria of pinealocytes, the AANAT/SNAT has been identified in the mitochondria of oocytes and the suitable substrate (acetyl CoA) availability for AANAT in mitochondria. In addition, the high level of melatonin is detected in the medium of cultured mitochondria. The protective effects of melatonin on mitochondria depend on its accumulation in these organelles. To achieve this, it requires an active melatonin transport against a concentration gradient. Melatonin mitochondrial carriers have been reported recently, and their levels in mitochondria were positively associated with mitochondrial melatonin concentration. An important protective mechanism of melatonin on mitochondria is that melatonin influences the mitochondrial membrane potential (Δψ). Melatonin blocks MPTP to preserve the Δψ under stressful conditions and activates the UCPs to slightly reduce the Δψ in normal condition. These activities are not in conflict with each other. Blockage of MPTP prevents the Δψ collapse and cellular apoptosis. Activation of UCPs reduces ROS formation because a slight lowering of Δψ accelerates the electron transportation and reduces electron leakage. Activation of UCPs seems not to reduce ATP production as expected. A potential mechanism is that the fewer leaked electrons under the UCP activation contribute their energy to ATP production. A balanced Δψ is ideal for the function of mitochondria. The detailed information as to the mechanisms is summarized in Figure 4. In addition to mitochondrial protection, melatonin also influences mitochondrial dynamics. The daily oscillations of mitochondrial functions as well as the morphology seem to fit well with the melatonin circadian rhythm. Melatonin reduces mitochondrial fission and increases their fusion, thereby preserving their normal function. Recently, it has been reported that melatonin modified mitophagy by either the enhancement or the reduction of this process, depending on conditions and cell types. The exact mechanisms require further investigation.
